# Supplementary material for: Causal Relationship between Aging and Anorexia Nervosa: A White-Matter-Microstructure-Mediated Mendelian Randomization Analysis
Source: Biomedicines. 2024 Aug 16;12(8):1874. doi: 10.3390/biomedicines12081874 (PMC11351342; doi:10.3390/biomedicines12081874)
Supplement: Supplementary file 1 [file biomedicines-12-01874-s001.zip › Supplementary Figure/Figure S8/Leaveoneout plot of MR analysis for TL to ubm-b-1473.pdf]

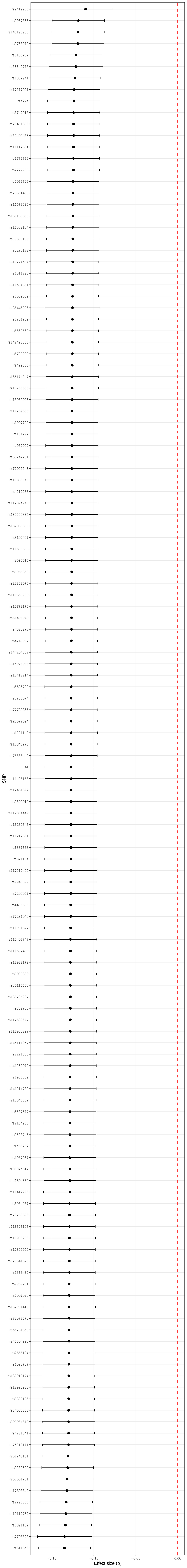

rs9419958
rs2967355
rs143190905
rs2763979
rs8105767
rs35640778
rs1332941
rs17677991
rs4724
rs5742915
rs78491606
rs59409453
rs11117354
rs6776756
rs7772289
rs2056726
rs75664430
rs11579626
rs150150565
rs11557154
rs28502153
rs2276182
rs10774624
rs1611236
rs11584821
rs6659669
rs35446936
rs6751209
rs6669563
rs142426306
rs6790988
rs429358
rs185174247
rs10768683
rs13062095
rs11769630
rs1907702
rs131797
rs932002
rs55747751
rs76065543
rs10805346
rs4616688
rs112394943
rs139669835
rs182059586
rs8102497
rs11699829
rs939916
rs9955360
rs28363070
rs116863223
rs10773176
rs61405042
rs4530278
rs4743037
rs144204502
rs16978028
rs12412214
rs6536702
rs3785074
rs77732866
rs28577594
rs1291143
rs10840270
rs76666449
All
rs11426156
rs12451892
rs9600019
rs117034449
rs13230646
rs11212631
rs6881568
rs871134
rs117512405
rs9940099
rs7209057
rs4498805
rs77231040
rs11991877
rs117407747
rs111527438
rs12932179
rs3093888
rs80116508
rs139795227
rs869785
rs117630647
rs111950327
rs145114957
rs7221585
rs41269079
rs1985369
rs141214782
rs10845387
rs6587577
rs7164950
rs2538745
rs450962
rs1957937
rs80324517
rs41304832
rs11412296
rs6054257
rs73730598
rs113525195
rs10905255
rs12369950
rs376641875
rs9878436
rs2282764
rs6007020
rs137901416
rs79977579
rs66731853
rs45604339
rs2555104
rs1023767
rs188918174
rs12925933
rs9398196
rs34550383
rs202034370
rs4731541
rs76219171
rs61748181
rs2230590
rs56061761
rs17803849
rs7790856
rs10112752
rs3891167
rs7705526
rs611646
SNP
-0.15
-0.10
-0.05
0.00
Effect size (b)
